# Subjective cognitive decline and cognitive leisure activity engagement in older adults: a mixed-methods study

**DOI:** 10.1093/geroni/igag088

**Published:** 2026-08-06

**Authors:** Emine Akbayrak, Philip A Powell, Sarah Barnes

**Affiliations:** Sheffield Centre for Health and Related Research, School of Medicine and Population Health, University of Sheffield, Sheffield, United Kingdom; Sheffield Centre for Health and Related Research, School of Medicine and Population Health, University of Sheffield, Sheffield, United Kingdom; Sheffield Centre for Health and Related Research, School of Medicine and Population Health, University of Sheffield, Sheffield, United Kingdom

**Keywords:** Health behavior, Lifestyle, Memory, Metacognition, Thematic analysis

## Abstract

**Background and Objectives:**

Subjective cognitive decline (SCD), self-perceived worsening in cognitive abilities without objective impairment, is an early marker of dementia linked to poorer mental health and reduced quality of life. Cognitive leisure activities (CLA) are low-cost, accessible behaviors that support cognitive functioning and well-being. However, evidence remains limited regarding the directionality, mechanisms, and domain-specific nature of the SCD–CLA relationship. This study provides novel mixed-methods evidence to inform early, person-centered intervention strategies.

**Research Design and Methods:**

A sequential explanatory mixed-methods design combined a cross-sectional survey of 255 older adults and semi-structured interviews with 20 individuals living with SCD in the United Kingdom. Quantitative data were analyzed using regression analyses, and qualitative data through thematic analysis. Results were integrated via a joint display.

**Results:**

Quantitative analyses showed higher SCD concerns were associated with lower CLA engagement, with specific activity types linked to distinct cognitive domains. Qualitative findings highlighted motivations, barriers, and strategies shaping engagement, including enjoyment, social connection, and emotional or health-related challenges. Integrated findings indicated potential reciprocal influences: cognitive concerns could motivate CLA engagement to maintain cognitive health, but SCD-related emotional distress and reduced confidence constrained participation. CLA did not statistically moderate the age–SCD association, yet participants reported perceived cognitive and emotional benefits.

**Discussion and Implications:**

These findings highlight the need for interventions that move beyond simple activity promotion to address emotional ambivalence, stigma, and contextual barriers to participation. Tailoring CLA opportunities to individual preferences and capacities may enhance engagement, cognitive wellbeing, and emotional resilience in later life.

Innovation and Translational Significance: This mixed-methods study provides novel, domain-specific insights into the relationship between cognitive leisure activities (CLA) and subjective cognitive decline (SCD) in older adults. Findings show patterns suggesting potential reciprocal influences in which SCD-related concerns can both motivate and limit engagement through emotional ambivalence, reduced confidence, and contextual barriers. Sustained CLA engagement may be supported through stigma-sensitive messaging, emotional reassurance, and practical assistance such as transport, mobility or technology support, and clear promotion of community opportunities. These findings can inform community programs, early cognitive health interventions, and clinical guidance in later life.

## Background and objectives

As populations age globally, maintaining cognitive health in later life has become a key public health priority (Mitchell & Walker, 2020). While some cognitive slowing is expected with normal aging, many older adults report concerns about their memory or thinking in the absence of objective impairment, a phenomenon referred to as subjective cognitive decline (SCD) (Jessen et al., 2014). SCD is recognized as a potential early marker of pathological decline and dementia (Wang et al., 2021) and is also linked to increased anxiety, depression, and reduced quality of life (Jenkins et al., 2019). These concerns often appear before clinical symptoms, making SCD a key target for early detection and prevention in cognitive aging research.

Recent estimates suggest that up to 40% of dementia cases may be preventable through the modification of life-course risk factors (De Looze et al., 2023). Among these, cognitive leisure activities (CLA), such as reading, playing games, or engaging in intellectually stimulating hobbies, are consistently associated with better cognitive outcomes and reduced risk of decline in later life (Mao et al., 2023; Park et al., 2014). Beyond direct cognitive benefits, engagement in these activities can also reduce stress and depressive symptoms, fostering psychological well-being that indirectly contributes to cognitive health (Kim et al., 2022). However, important gaps remain, particularly regarding the relationship between SCD and CLA engagement. A recent systematic review (Akbayrak et al., 2024) found that while higher CLA engagement was generally linked to fewer SCD symptoms, findings were inconsistent due to differences in study design and outcome measures. Most studies focused on memory-related concerns, overlooking other cognitive domains such as language and executive function. Moreover, limited research has examined how engagement in different types of CLA relates to total and domain-specific SCD concerns. The review also identified a lack of qualitative research exploring older adults’ subjective experiences with CLA, specifically, the motivations and barriers to participation and the perceived cognitive or emotional benefits of different CLA types.

These limitations highlight the need for more comprehensive, multi-method approaches to understand how and why older adults with SCD engage—or disengage—from CLA. While quantitative studies can establish statistical associations between SCD and CLA engagement, qualitative research offers insights into the personal meanings, contextual influences, and lived experiences that shape these behaviors. Therefore, to address these gaps, this study adopted a sequential explanatory mixed-methods design. Quantitative analyses examined associations between overall and domain-specific SCD and CLA engagement frequency and variety. We also explored the potential moderating role of CLA on the relationship between age and SCD, as well as links between specific types of CLA and domain-specific SCD concerns. The qualitative component explored older adults’ lived experiences with CLA, focusing on motivations, barriers, and strategies for engagement. Consequently, this study aims to (a) quantitatively examine the relationship between SCD and CLA engagement in older adults, (b) understand participants’ qualitative experiences and perspectives on CLA participation, and (c) integrate quantitative and qualitative findings to provide a comprehensive understanding of the SCD–CLA relationship.

## Research design and methods

### Research design and participants

This study used an explanatory sequential mixed-methods design, in which quantitative data were first collected to examine associations between CLA engagement and SCD, followed by qualitative interviews to explore the underlying mechanisms and contextual factors. Quantitative analyses alone cannot capture the subjective, emotional, and motivational dimensions of CLA engagement and SCD; the qualitative phase therefore provided insight into participants’ lived experiences, offering a more comprehensive understanding of how and why they are related. A prior systematic review identified a lack of qualitative research in this area (Akbayrak et al., 2024), further motivating the inclusion of a qualitative component. Both phases were equally emphasized. Ethical approval was obtained from the University of Sheffield Research Ethics Committee, and all procedures followed institutional standards.

For the quantitative phase, community-dwelling adults aged ≥60 years residing in the United Kingdom were eligible if they reported no diagnosed cognitive disorders (e.g., dementia or Alzheimer’s disease) and no mental health conditions (e.g., major depression or schizophrenia). Eligibility was confirmed using screening questions at survey entry. Participants were recruited between January and June 2024 using a mixed-mode convenience sampling strategy. Data were collected via online (Qualtrics) and paper-based surveys distributed through charitable organizations and community groups. These organizations shared the survey via mailing lists, social media, and in-person distribution at community venues. Survey packs, including information sheets and consent forms, were also provided for on-site and postal distribution. Written informed consent was obtained digitally or in paper form. The minimum required sample size was calculated using G*Power, based on a small effect size (*f²* = 0.048) from an analogous previous study (Lee et al., 2020), with *α*  =  0.05, power = 0.80, and five predictors, yielding a minimum target of 166 participants. In total, 267 individuals were initially recruited. Ten participants were excluded for not meeting the inclusion criteria, including incomplete surveys (*n *= 3), being under the age of 60 (*n *= 2), reporting mental health issues (*n *= 3), or reporting diagnosed cognitive disorders (*n *= 2), yielding a final quantitative sample of 255 (46 online, 209 paper-based).

Participants for the qualitative phase were drawn from the quantitative sample. Individuals who completed the survey, expressed interest in further participation, and scored at or above the established cutoff score of 7 on the Subjective Cognitive Decline Questionnaire (SCD-Q), a threshold used to distinguish individuals reporting SCD concerns, were eligible for interviews. Purposive sampling was used to ensure variation in both the severity of SCD concerns and the levels of CLA engagement. A target sample of 20 participants was set to ensure sufficient depth and diversity of perspectives, with thematic saturation monitored throughout data collection. To facilitate richer exploration of lived experiences, individuals with more pronounced SCD concerns were prioritized within the eligible pool. To ensure variation in experiences across activity engagement levels, participants were purposively sampled based on their Cognitive Leisure Activity Scale (CLAS) scores. As there are no established cutoff points for categorizing CLA engagement levels, the sample mean score (*M *= 31) was used as a pragmatic threshold. Participants scoring above the sample mean were classified as having higher CLA engagement, whereas those scoring below the mean were classified as having lower CLA engagement. Ten participants were then selected from each group to ensure variation in activity engagement within the qualitative sample.

### Data collection

#### Survey measures

The survey comprised three components: (a) a sociodemographic questionnaire, (b) the SCD-Q (Rami et al., 2014), and (c) the CLAS (Galvin et al., 2021). The sociodemographic questionnaire collected participants’ age, gender, education level, and marital status. The SCD-Q includes 24 dichotomous (yes/no) items assessing perceived changes in memory (11 items), language (6 items), and executive functioning (7 items), divided into MyCog (self-report) and TheirCog (informant) sections. Only the MyCog section was administered to examine participants’ own perceptions of cognitive changes. MyCog reliably distinguishes individuals reporting SCD from cognitively healthy controls using an established cutoff of seven items, with excellent internal consistency (*α* = .90), good sensitivity (83%), specificity (87%), and overall discrimination (area under the curve [AUC] = .91; Rami et al., 2014); internal consistency in this sample was *α* = .89. The SCD-Q was selected because it captures perceived cognitive changes across multiple domains, rather than relying on single-item questions or focusing solely on memory complaints (Ibnidris et al., 2022). It assesses gradual, self-perceived changes over the past 2 years rather than ability-based functional performance, is practical and concise, uses a clear response format, and allows SCD concerns to be analyzed continuously (Rabin et al., 2023). The CLAS is a brief, validated instrument quantifying engagement in cognitively stimulating leisure activities over the past year (Galvin et al., 2021). It comprises 16 items reflecting common activities (e.g., reading, puzzles, musical instruments) and uses a 6-point Likert-type frequency scale to capture both frequency and diversity of engagement, with higher scores indicating greater participation. Developed to address the lack of standardized measures specifically targeting CLA in older adults, the CLAS demonstrates acceptable internal consistency (*α* = .73; *α* = .67 in this study). Compared with other instruments, it is brief, easy to administer, focuses specifically on cognitive leisure rather than activities of daily living or physical activity, and allows quantification of engagement over a defined period (Galvin et al., 2021).

#### Semi-structured interviews

Semi-structured interviews were conducted to explore older adults’ motivations for, barriers to, and strategies supporting engagement in CLA. The interview guide was informed by literature on CLA and SCD (e.g., systematic reviews; Akbayrak et al., 2024) and findings from the quantitative phase assessing SCD concerns and CLA engagement, which guided question development. For example, questions probed perceived cognitive benefits of CLA (e.g., “Do you feel that engaging in these activities helps with your memory or thinking skills?”) to examine patterns suggested by the quantitative data. To promote accessibility and open discussion, lay terminology such as “brain health activities” was used instead of technical language. Participants were first asked to describe perceived cognitive changes and their impact on daily functioning, followed by a discussion of activities undertaken. Subsequent questions explored motivations, barriers, and strategies for maintaining or increasing engagement. Initial contact was made by email with an information sheet; if no response was received within 1 week, participants with a phone number were contacted by telephone. Two participants could not be reached, one withdrew shortly before the interview, and two declined; replacements were invited to maintain sample diversity. Interviews were conducted individually, either face-to-face or via video conferencing, according to preference. Written informed consent was obtained (paper for face-to-face; electronic via Qualtrics for online interviews), and interviews were audio-recorded with permission. Interviews lasted between 30 min and 1 hr and 20 min (*M *= 45 min) and were conducted between September and November 2024. Variation in length reflected differences in participants’ willingness to elaborate and perceived relevance of SCD to their engagement. The full interview guide is provided in Supplementary Material Section 1.

### Analysis

For the quantitative study, analyses were conducted using IBM SPSS Statistics (Version 29). Data were cleaned and scored, with no missing data, and descriptive statistics summarized participant characteristics and key variables. Due to non-normality, Spearman correlations examined associations between age, CLA engagement (variety and frequency), and total and domain-specific SCD scores. Mann–Whitney *U* tests compared SCD scores across categorical variables (education, marital status, gender). Linear regression included only significantly correlated variables to assess independent predictors of total and domain-specific SCD, with bootstrapped 95% confidence intervals (1,000 resamples) for robustness. Moderated regression tested whether overall CLA engagement moderated the relationship between age and SCD, examining its protective role. CLA engagement was analyzed both as a continuous total score and at the individual activity level to identify activities most closely associated with lower SCD concerns. For the activity-level analysis, responses were recoded into a binary variable (0 = *Never*, 1 = *Any engagement*). The original scale assessed frequency on a 6-point Likert scale (Never, Several times per year, Several times per month, Once per week, Several times per week, Daily). For the binary coding, “Never” responses were coded as 0, whereas all other response options were coded as 1. This sequential approach allowed comprehensive evaluation of associations between CLA participation and SCD while accounting for sociodemographic factors and potential moderation effects.

For the qualitative study, the interview data were analyzed using a coding reliability approach to thematic analysis (Braun & Clarke, 2022). This approach was selected because the three broad thematic domains were determined a priori, and the analytic process involved multiple coders, both of which are consistent with a coding reliability approach to thematic analysis (Braun & Clarke, 2022). Codes were developed inductively from the data within each predefined domain (Joffe, 2011). All interviews were audio-recorded, transcribed verbatim, anonymized, and imported into NVivo (version 14). The first author read all transcripts multiple times before coding began. A subset of four transcripts was then independently coded by the first author, two coauthors, and an institutional colleague, each generating codes within the three thematic domains. The research team included researchers with expertise in gerontology, as well as qualitative and mixed-methods research experience. No formal statistical measure of intercoder reliability was calculated. Instead, to support coding consistency, an intercoder consistency approach was adopted (O’Connor & Joffe, 2020). Coded outputs from the four transcripts were systematically compared across coders to identify areas of agreement and disagreement. Coding discrepancies were discussed and resolved through negotiated consensus, with code definitions refined where necessary. Following this process, the first author applied the agreed coding approach to the remaining transcripts. A code frequency grid was used to track when no new codes emerged across interviews, consistent with the coding reliability thematic analysis (Braun & Clarke, 2021; see Supplementary Section 2).

### Integration of findings

Integration occurred through joint displays comparing quantitative and qualitative results to identify convergences, divergences, and complementarities (Guetterman et al., 2015). This enabled a comprehensive understanding of the SCD–CLA relationship beyond single-method insights. The study followed established reporting guidelines: Strengthening the Reporting of Observational Studies in Epidemiology (STROBE) (Vandenbroucke et al., 2007) for the quantitative component (Table S3.1), Standards for Reporting Qualitative Research (SRQR) (O’Brien et al., 2014) for the qualitative component (Table S3.2), and Good Reporting of A Mixed Methods Study (GRAMMS) (O’Cathain et al., 2008) for the mixed-methods design (Table S3.3).

## Results

### Quantitative findings

The final sample comprised 255 participants aged 60–95 years (*M *= 76.9, *SD *= 8.2); 74.9% were women, 80.4% had no university degree, and 41.6% were married. Overall, 51% of participants reported experiencing SCD, with a mean total SCD score of 7.17 (*SD *= 5.56), while mean CLA engagement frequency and variety scores were 31.38 (*SD *= 9.01) and 9.97 (*SD *= 2.72), respectively (Table 1).

**Table 1:** Descriptive characteristics of research sample (*N *= 255).

Variable	Full sample			Qualitative sample	
	*N* (%)	*M* (*SD*)	Range	*M* (*SD*)	Range
**Gender**					
Man	64 (25.1)				
Woman	191 (74.9)				
**Education level**					
Degree	50 (19.6)				
No degree	205 (80.4)				
**Marital status**					
Married	106 (41.6)				
Not married	149 (58.4)				
**Presence of SCD**					
Yes	130 (51.0)				
No	125 (49.0)				
**Age (years)**		76.94 (8.21)	60–95	74.48 (7.82)	60–85
**SCD total**		7.17 (5.56)	0–24	13.24 (3.53)	7–20
**SCD-memory**		2.70 (2.60)	0–11	5.13 (2.21)	2–10
**SCD-language**		2.44 (1.78)	0–6	3.45 (1.24)	1–5
**SCD-executive function**		2.04 (1.95)	0–7	3.32 (1.62)	1–7
**CLA engagement frequency**		31.38 (9.01)	10–59	33.47 (9.17)	20–55
**CLA engagement variety**		9.97 (2.72)	3–16	11.81 (2.31)	7–14

*Note.* CLA = cognitive leisure activity; *N* = number of participants; SCD = subjective cognitive decline.

No significant differences in total or domain SCD scores were found by gender, education, or marital status (all *p* > .30; see Supplementary Section 4). Higher CLA engagement, both in terms of frequency and variety, was modestly but significantly associated with lower SCD concerns across most domains (*r *= −.144 to −.186, *p* < .05; Table 2). Age was positively but weakly associated with SCD scores across most domains (*r* = .151 to .208, *p* < .05). For the language domain, CLA engagement frequency showed a weak negative correlation, whereas CLA variety and age were not significantly associated (Table 2).

**Table 2:** Correlation coefficients among SCD scores, CLA variables, and age.

Variables	1	2	3	4	5	6	7
1. CLA engagement variety	1						
2. CLA engagement frequency	.823**	1					
3. Age	−.152*	−.063	1				
4. SCD total	−.163**	−.186**	.174**	1			
5. SCD-memory	−.168**	−.182**	.151*	.910**	1		
6. SCD-language	−.104	−.159*	.122	.866**	.692**	1	
7. SCD-executive function	−.144*	−.146*	.208**	.842**	.654**	.631**	1

*Note.* CLA = cognitive leisure activity; SCD = subjective cognitive decline.

* *p* < .05.

** *p* < .01 (two-tailed).

Linear regression examined the extent to which age and CLA engagement predicted total SCD concerns (Table 3). CLA engagement variety was excluded from the model due to high correlation with engagement frequency (*r* = .823, *p* < .01), to avoid multicollinearity. Age was included as a covariate because it showed a significant bivariate association with SCD, whereas other demographic variables did not. The model was statistically significant, explaining 6.2% of the variance in total SCD (*F*(2, 252) = 9.394, *p* < .001), with age positively (*β* = .169, *p* = .006) and CLA engagement negatively (*β* = −.191, *p* = .002) predicting total SCD. Similar patterns were observed for memory and executive domains, whereas age showed only a marginal association with the language domain (*β* = .116, *p* = .061; see Supplementary Section 5).

**Table 3:** Linear and moderated regression analyses predicting total SCD score.

Variable	*b*	*SE*	*β*	*t*	*p*	95% CI
**Linear model**						
Constant	7.182	.337	–	21.283	<.001	[6.52, 7.93]
Age	.114	.041	.169	2.770	.006	[.03, .20]
CLA engagement frequency	−.118	.038	−.191	−3.136	.002	[−.12, −.04]
Model summary	*R*² = .069, adjusted *R*² = .062, *F*(2, 252) = 9.394, *p* < .001.					
**Moderated models**						
**Model 1**						
Constant	7.182	.337	–	21.283	<.001	[6.54, 7.87]
Age	.114	.041	.169	2.270	.006	[.02, .19]
CLA engagement frequency	−.118	.038	−.191	−3.136	.002	[−.12, −.04]
Model summary	*R*² = .069, adjusted *R*² = .062, *F*(2, 252) = 9.394, *p* < .001					
**Model 2**						
Constant	7.198	.339	–	21.243	<.001	[6.53, 7.88]
Age	.118	.042	.174	2.825	.005	[.03, .19]
CLA engagement frequency	−.120	.040	−.195	−3.179	.002	[−.20, −.04]
CLA engagement frequency × age	.003	.005	.039	.629	.530	[−.09, .01]
Model summary	*R*² = .071, Δ*R*² = .001, *F*(3, 251) = 6.380, *p* < .001; Δ*F*(1, 251) = .396, *p* = .530.					

*Note.* 95% confidence intervals (CI) calculated using 1,000 bootstrap samples. CLA = cognitive leisure activity; SCD = subjective cognitive decline.

Moderated regression analysis was conducted to examine whether CLA engagement frequency buffered the association between age and total SCD score (Table 3). Age was selected as the predictor to be moderated because prior analyses indicated it was significantly associated with SCD, whereas other demographic variables were not. All predictors were mean-centered before computing the interaction term, and bootstrapping was applied, as described above. In Model 1, age and CLA engagement frequency significantly predicted total SCD, explaining 6.2% of the variance (*R*² = .069, *F*(2,252) = 9.394, *p* < .001). In Model 2, the Age × CLA engagement interaction was non-significant (*β* = .039, *p* = .530; Δ*R*² = .001, Δ*F*(1,251) = .396), indicating no evidence of a moderating effect. Domain-specific analyses yielded comparable non-significant interaction effects (see Supplementary Section 6).

Correlations between specific CLA types and SCD scores (*r*; Table 4) revealed several significant negative associations that varied by domain. Exercise demonstrated the strongest and most consistent association with lower total and domain-specific SCD scores. Engagement in crossword, jigsaw, and sudoku activities was also associated with reduced total, memory, and executive SCD concerns. Reading was associated with lower total, memory, and language-related SCD concerns, whereas attending theater or concerts was associated with lower total, memory, and executive SCD. Chess, checkers, or backgammon, and attendance at clubs or group activities were specifically associated with fewer memory concerns only. Other assessed activities (e.g., volunteering, socializing, and arts and crafts) showed no significant associations.

**Table 4:** Correlations between specific CLA types and SCD scores.

CLA types	Total SCD score		Memory score		Language score		Executive score	
	*B*	*p*	*B*	*p*	*B*	*p*	*B*	*p*
Chess/Checkers/Backgammon	−.116	.063	−**.156**	**.013**	−.054	.387	−.082	.192
Crossword/Jigsaw/Sudoku	−**.161**	**.010**	−**.169**	**.007**	−.114	.069	−**.148**	**.018**
Attending a club or group activity outside the home	−.120	.056	−**.154**	**.014**	−.068	.281	−.066	.294
Reading a newspaper, book, or magazine	−**.151**	**.016**	−**.188**	**.003**	−**.141**	**.025**	−.028	.653
Attending the theater, concert, or symphony	−**.148**	**.018**	−**.150**	**.016**	−.071	.260	−**.152**	**.015**
Exercise (any type)	−**.230**	**<.001**	−**.197**	**.002**	−**.191**	**.002**	−**.228**	**.001**

*Note.* CLA = cognitive leisure activity; SCD = subjective cognitive decline. Bold values indicate statistically significant correlations (*p* < .05).

### Qualitative findings

Twenty participants (16 women, 4 men) aged 60–85 years (*M *= 74.5, *SD *= 7.8) took part in the qualitative interviews (Table 1). Participants’ SCD-Q scores ranged from 7 to 20 (*M *= 13.2, *SD *= 3.5), and CLAS scores ranged from 20 to 55 (*M *= 33.5, *SD *= 9.2).

Thematic analysis identified three main themes: motivations, barriers, and strategies. Participants highlighted personal and social motivations (e.g., enjoyment, achievement, social engagement, cognitive maintenance); barriers (e.g., perceived inability, emotional resistance, health issues, time constraints); and strategies (e.g., accessibility and awareness, social support, tailored guidance).

#### Motivations for CLA engagement

Participants described a range of motivations for engaging in CLA, shaped by intrinsic enjoyment, perceived cognitive and emotional benefits, and opportunities for social connection, which are visually summarized in Figure 1. For many, enjoyment emerged as a central driver, with activities chosen based on personal interest, providing immersion, mental escape, and sustained participation through pleasure and fulfillment. One participant emphasized engagement was “*for fun*” rather than an obligation. Alongside enjoyment, several participants viewed CLA as a proactive strategy to preserve cognitive functioning, describing mental activity as “keeping the brain going” and resisting feared deterioration. Some reported tangible cognitive benefits in language use and concentration: “*The more that I use language, the more I feel that I will actually remember the words, if I use recent words that I would be using, I will remember but if I haven’t used them for a while, I will forget them. […] I do feel that that activity has kept me going, it’s definitely been a benefit, […] whenever I’m interested in something, my brain can work well.*” This suggests that CLA functioned not merely as a passive activity but as an active, self-directed cognitive strategy: one directly shaped by awareness of SCD and a desire to counteract perceived decline. Even when changes were difficult to assess objectively, the belief that CLA could protect against decline offered reassurance and a sense of control.

**Figure 1:**
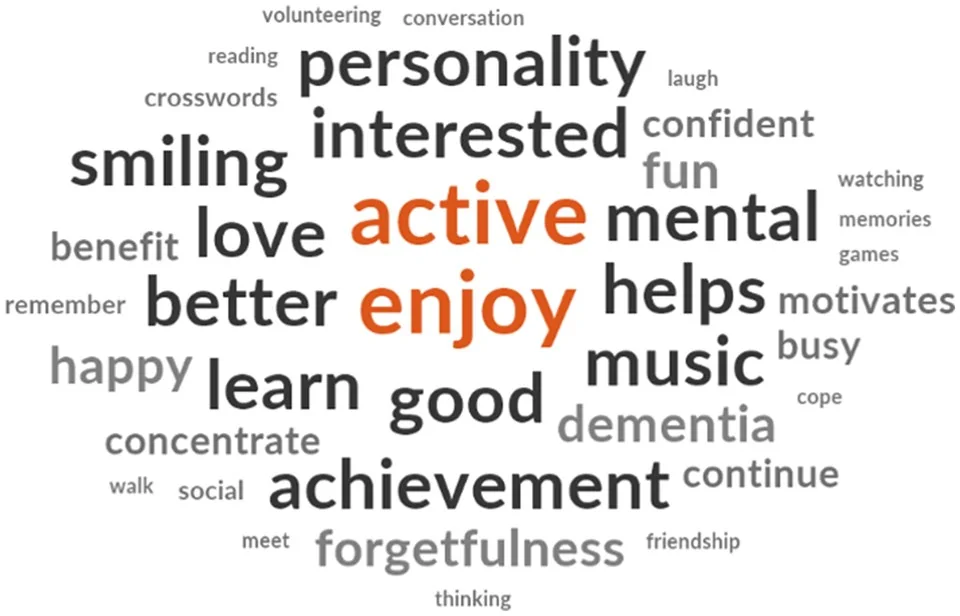
Motivations for cognitive leisure activity engagement.

Engagement in CLA also supported confidence and self-efficacy. Remaining mentally active reassured individuals of their ability to manage forgetfulness, while learning new skills fostered accomplishment and growth. One participant described feeling “*more confident about managing forgetfulness*,” illustrating how CLA engagement may support psychological adaptation to SCD by reinforcing a sense of agency and cognitive resilience—dimensions not captured by symptom-focused SCD measures. Social connection represented another prominent motivation, with group activities providing belonging and emotional well-being. As one participant reflected, “*I do a dance fit once a week, […] I do go to these other friendship lunches, because that’s meeting people and making you feel better that you’ve seen other people.*” For some, social contact was a primary motivator shaping activity choices and broader life decisions, including relocation to more socially connected communities: “*I’m hoping to move to [neighbourhood] because I know a few people there, and there’s so much community you can join in with, and it just keeps you going, I think that’s the biggest factor, you know, looking forward to the next thing.*” The prominence of social motivation among widowed and socially isolated participants suggests that loneliness and heightened SCD concerns may reinforce one another, with CLA serving as a key mechanism for maintaining social connectedness and emotional wellbeing. Notably, participants’ motivations varied according to SCD concerns and personal circumstances. Preventing cognitive decline was more prominent among those with higher SCD concerns, while maintaining social engagement was emphasized by widowed or living-alone individuals who often linked loneliness to emotional distress and heightened SCD complaints. This variation indicates that motivations for CLA are shaped by individual SCD profiles and psychosocial context, underscoring the need for person-centered approaches to supporting engagement.

### Barriers to CLA engagement

Participants described multiple, interconnected barriers to CLA engagement: cognitive difficulties, psychological and emotional challenges, health limitations, and contextual constraints such as caregiving responsibilities (Figure 2). A key barrier was the perceived inability to engage due to memory difficulties, mental fatigue, and increased cognitive effort. Previously effortless tasks now caused frustration and heightened awareness of decline. One participant described how formerly enjoyable activities had become a reminder of worsening ability: “*They show you that you’re getting worse. […] gradually over the years, the puzzles I used to be able to do the really hard ones, and now I can’t actually […] it is demanding. […] like doing crossword, Sudoku reminds me that my brain functions are getting worse.*” Some withdrew entirely, while others adapted by simplifying tasks or choosing less demanding alternatives. This variability suggests that the same cognitive concerns can prompt either withdrawal or adaptive coping, depending on individual psychological resources and resilience. Psychological and emotional barriers further constrained participation. Reduced confidence, fear of failure, and anxiety from prior negative experiences undermined motivation. One participant described losing confidence after becoming disoriented: “*I don’t go on the bus into town very often. I think I lost confidence when it was covid. […] I couldn’t remember the number of the bus to get back home, and I couldn’t find bus stop, and I got really panicked. […] ever since then, I’ve been very worried of being on my own getting lost.*” Embarrassment or fear of judgment due to cognitive lapses made demanding or social activities daunting. Social stigma related to aging and personal traits such as reluctance to try new experiences and preference for familiar routines reinforced avoidance. Together, these factors created a cycle in which negative emotions reduced confidence and motivation, limiting sustained CLA engagement.

**Figure 2:**
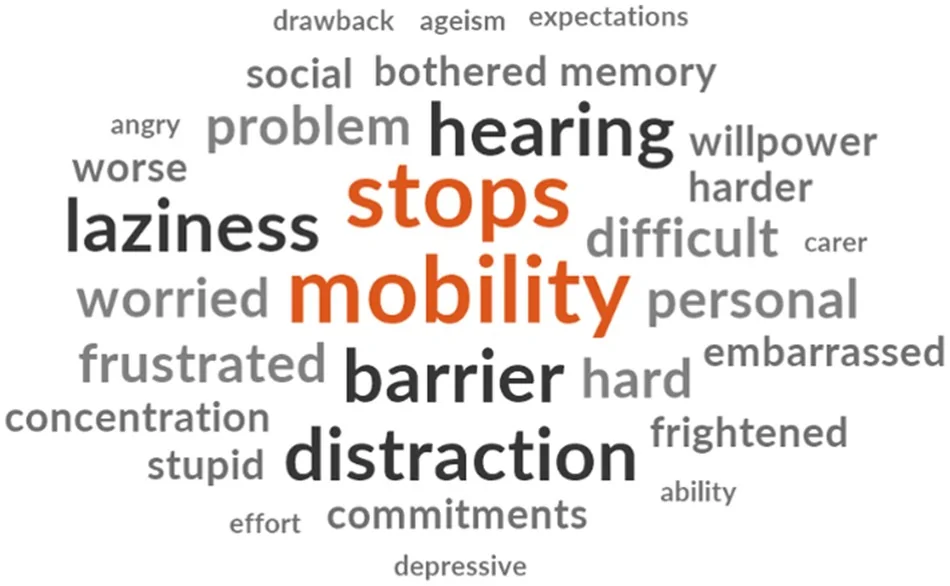
Barriers to cognitive leisure activity engagement.

Health-related limitations were significant barriers. Chronic conditions, sensory impairments, and mobility challenges restricted participation directly and indirectly by promoting social withdrawal and lowering motivation. Hearing problems were particularly impactful: “*I have a hearing problem, […] if you have a hearing problem, I feel what’s the point in going out? So then if you don’t go out, you’re not keeping your brain active, and you’re maybe going to fall into this habit of making the memory worse […] I’m not going out because I can’t hear and I just look foolish in front of people.*” Pain from arthritis or serious illness disrupted routines and confidence, while mobility limitations restricted independent access to community-based CLA: “*Well, for me, living here, it was quite a long walk […] I can’t do that now […] So actually, physically, not being as mobile, that’s a big drawback.*” Caregiving responsibilities further limited time and energy for personal activities. One participant explained: “*Because I’m a caregiver, that’s a big barrier, a lot of my time is spent looking after my husband, and I don’t have as much time to do my own. […] I am missing out on exercise, not just because of my physical disability but because of my caring role.*” Overall, barriers to CLA reflect a complex interplay of internal challenges and external constraints. Health issues and caregiving demands were more common among those with lower engagement, while internal barriers such as fear, low confidence, and reduced motivation occurred across all levels. Individual resources to manage these challenges appear to influence continued participation. These patterns suggest that barriers are shaped not only by cognitive and physical capacity but also by the broader psychosocial context of aging with SCD, highlighting the need for interventions addressing both structural and emotional dimensions of engagement.

#### Strategies to support CLA engagement

Participants shared strategies to support CLA, especially for those facing social isolation or structural barriers. Key themes included raising awareness, improving accessibility, fostering social encouragement, and providing empathetic professional support. Improving availability, accessibility, and awareness of CLA opportunities was viewed as fundamental. Many participants were unaware of existing activities, highlighting the need for clearer communication, better advertising, and improved coordination among community organizations. One participant noted: “*Just really making sure everybody knows about these things that are there.*” Practical accessibility, particularly transport, was also essential, as limited mobility could prevent engagement. Digital resources, such as apps and online quizzes, were suggested as complementary options to provide cognitive stimulation and reduce access barriers. These findings suggest that structural barriers, rather than a lack of motivation, may be a primary driver of low CLA engagement among older adults with SCD, pointing to the importance of environmental and systemic interventions. Social encouragement was another key facilitator. Support from family, friends, and community members helped participants overcome self-doubt, low motivation, and anxiety about cognitive difficulties. Invitations, reminders, and shared experiences fostered belonging and made participation less intimidating. One participant described: “*It’s a series of tiny breakthroughs. But you need help. You need somebody to stimulate you, because you’ve got the fears.*” This support was especially important for those with memory or confidence challenges, helping them initiate and sustain engagement.

Participants also highlighted the need for empathetic, person-centered professional guidance. Some felt professionals struggled to understand cognitive difficulties fully, limiting effective support. One participant explained: “*I think that although professionals try to understand the elderly, it is difficult for them to put themselves in our shoes and approach them because they have not experienced the same things, they cannot fully understand what is happening, what you are going through.*” Empathy, patience, and validation were viewed as essential for reducing embarrassment, building trust, and encouraging participation. Together, participants emphasized that effective CLA support combines structural solutions, social encouragement, and empathetic professional guidance, highlighting the importance of accessible, inclusive, and person-centered approaches to help older adults with SCD remain cognitively active.

### Integrated findings

The integrated analysis (Table 5) suggests reciprocal influences between SCD and CLA engagement, whereby SCD concerns may shape engagement while participation in CLA is associated with levels of SCD concern. Quantitatively, higher and more varied CLA participation was associated with lower total and domain-specific SCD concerns. Qualitative findings help explain this relationship by revealing contrasting motivational pathways through which SCD experiences shape engagement. Memory and concentration difficulties often reduced confidence and motivation through negative emotional responses, leading some individuals to withdraw from cognitively demanding activities, whereas concern about cognitive decline motivated others to maintain or increase participation as a strategy to preserve cognitive health. Sustained engagement was further facilitated by external supports, including accessible activities, transport, social encouragement, and empathetic professional guidance, positioning CLA not only as a cognitive resource but also as an outcome shaped by subjective perceptions of decline and available supports. Quantitative analyses identified age as the strongest sociodemographic predictor of SCD. Qualitative findings expand on this pattern by showing that adults aged 80 and above, particularly those living alone or widowed, often experienced greater emotional distress linked to heightened cognitive concerns. These experiences shaped engagement differently: for some, distress and reduced confidence limited participation, whereas for others, similar concerns encouraged engagement to maintain social connection, daily structure, and well-being.

**Table 5:** Joint display of integrated findings.

Key insight	Quantitative findings	Qualitative findings	Integrated interpretation
Relationship between CLA engagement and SCD	Higher CLA engagement frequency was significantly associated with lower total SCD scores and lower domain-specific concerns (e.g., memory, language, and executive function), controlling for age.	SCD symptoms (e.g., memory lapses, concentration difficulties) sometimes reduced motivation and confidence for CLA, but concern about decline also motivated engagement. Social encouragement, accessible activities, transport, and empathetic professional support facilitated continued CLA engagement.	Greater CLA engagement may be theorized to protect against SCD, but symptoms often lower motivation and confidence through negative emotions (e.g., fear, shame, frustration), leading to withdrawal, especially when combined with ageism and stigma. Yet, these same fears can also motivate CLA as a self-driven effort to maintain cognitive health. Social and environmental supports play a complementary role in sustaining CLA participation.
Socio-demographic influences	Older age was significantly associated with higher total SCD, memory, and executive function concerns, while gender, education, and marital status showed no significant differences across total or domain-specific SCD scores.	Older participants, especially those aged 80+, reported experiencing greater emotional distress and reduced CLA engagement. Living alone was perceived to contribute to feelings of loneliness and heightened concerns about cognitive decline, but also motivated CLA engagement to preserve social contact.	Although quantitative analyses show limited effects of sociodemographic factors on SCD (except age), qualitative findings highlight that older age and being unpartnered may shape emotional and cognitive experiences, with social connections serving as key motivators for CLA engagement.
Moderating role of CLA engagement	CLA engagement did not significantly moderate the relationship between age and total or domain-specific SCD (no statistical protective effect).	CLA engagement was perceived to enhance cognitive functioning (e.g., memory, concentration, word recall, language fluency) and foster emotional well-being, boosting confidence, self-efficacy, and motivation to remain cognitively active.	There was no significant moderation effect of CLA on the age–SCD relationship, but qualitative data suggest a perceived buffering role of CLA, while the social dimension (e.g., structure, connection, reduced isolation) acted as a motivational and protective factor.
Activity-specific associations with SCD	Specific activities were differentially associated with SCD domains. Puzzles → lower total SCD, memory, & executive function concerns Reading → lower total, memory, & language concerns Playing chess, checkers, or backgammon/Participating in clubs/group activities → fewer memory concerns Attending theater, concerts, or symphonies → lower total, memory, & executive concerns	Puzzles and reading sometimes heightened awareness of cognitive decline, causing frustration, but many continued for mental stimulation and routine. Group-based CLA (e.g., knitting, bridge, dance) supported social engagement and reduced isolation. Volunteering provided purpose, social connection, and a sense of cognitive competence. Music was described as cognitively stimulating and memory-enhancing, often likened to a mental “revision” exercise.	Quantitative data show that a greater variety of CLA is associated with lower SCD, but qualitative findings reveal a complex picture. Some cognitively demanding activities increased awareness of decline, while social and group activities promoted engagement, emotional support, and reduced isolation. Other CLA (e.g., volunteering, music) offered perceived cognitive and emotional benefits despite lacking statistical associations. CLA engagement is multidimensional, supporting cognitive, social, and emotional well-being beyond what is captured quantitatively.

*Note.* CLA = cognitive leisure activity; SCD = subjective cognitive decline.

Although CLA engagement did not significantly moderate the age–SCD relationship quantitatively, qualitative findings indicated that participants perceived CLA as beneficial, describing improvements in memory, concentration, word retrieval, and language fluency alongside increased confidence and motivation. This discrepancy suggests that the benefits of CLA may operate through psychological and emotional processes, such as perceived cognitive control, emotional resilience, and sense of purpose, rather than emerging as a statistically detectable moderation effect. This may reflect a limitation of SCD measures, which primarily capture self-reported cognitive symptoms and may not fully account for the broader psychological experience of cognitive aging. Activity-level findings further extend this interpretation. Quantitatively, puzzles, reading, and group activities were associated with lower SCD concerns. Qualitative accounts, however, revealed that cognitively demanding activities could sometimes heighten awareness of decline and generate frustration. At the same time, activities such as volunteering, music engagement, and socializing, although not always statistically associated with SCD, were valued for their combined cognitive, emotional, and social benefits, providing purpose, belonging, and mental stimulation. Together, these findings highlight the multidimensional role of personally meaningful CLA in supporting cognitive and emotional well-being in later life and underscore the need for more comprehensive approaches to capturing the psychological dimensions of cognitive aging.

## Discussion and implications

This mixed-methods study examined the interplay between SCD and CLA engagement in later life. Integrated findings indicated patterns suggesting potential reciprocal influences and context-dependent effects: higher CLA frequency and variety were generally linked to fewer cognitive concerns, yet self-perceived decline sometimes reduced motivation to participate. Age emerged as a key factor influencing SCD, with older adults reporting greater emotional distress and lower engagement. Although quantitative analyses did not show a moderating effect of CLA on the age–SCD relationship, qualitative insights suggested that participation fostered cognitive stimulation, confidence, and emotional resilience. Overall, CLA appeared to function less as a direct cognitive protector and more as a psychological and social resource supporting adaptation to perceived cognitive changes.

Previous research has largely focused on the unidirectional influence of CLA on cognitive outcomes, showing that frequent participation is associated with better cognitive health (Lv et al., 2025; Sposito et al., 2015). The quantitative findings of the current study reaffirm this trend, though the association was modest (explaining 6.2% of variance in SCD). This likely reflects the multifactorial nature of SCD, in which CLA represents only one of many contributing factors alongside cognitive, psychological, and contextual influences. It may also reflect limitations of SCD measures, which primarily capture self-reported cognitive symptoms and may not fully account for process-level cognitive benefits described by participants, such as mental activation during challenging activities, improved word retrieval through regular language use, and perceived memory benefits from music engagement. Many participants described fear of cognitive decline and a desire to “*stay sharp*” as key motivators for maintaining or increasing involvement, consistent with prior research showing that perceived threat can prompt cognitively proactive behaviors (Parikh et al., 2016; Rotenberg et al., 2020). However, the same symptoms, such as forgetfulness, word-finding difficulties, or attention lapses, sometimes led to withdrawal due to embarrassment, fear of judgment, or feelings of inadequacy. These reactions mirror findings that frustration, embarrassment, and negative self-image are common responses to memory problems, often reducing participation in cognitively or socially demanding activities (Akbayrak et al., 2024; Hill et al., 2018). Altogether, these findings indicate that CLA engagement is shaped not only by cognitive capacity but also by emotional and motivational processes, highlighting the need to conceptualize this relationship as dynamic, with potential reciprocal influences.

Sociodemographic factors like age and marital status are commonly examined in relation to SCD in later life (Jessen et al., 2020; Ma et al., 2024). Consistent with prior research, both quantitative and qualitative findings indicated that older participants, particularly those aged 80 and above, reported greater SCD concerns accompanied by emotional distress, which could reduce CLA engagement (Akbayrak et al., 2024). Age-related neurobiological changes and cognitive slowing may increase the perceived effort of cognitively demanding activities (Kirk, 2024), as participants described difficulties sustaining attention or completing tasks that were previously manageable. Although marital status was not a significant predictor in quantitative analyses, qualitative accounts suggested that unpartnered participants often experienced heightened cognitive concerns linked to loneliness and reduced daily cognitive stimulation. The absence of routine conversations and shared problem-solving limited opportunities for mental engagement, with some participants noting that even minor spousal interactions had previously helped keep their minds active. These experiences align with evidence that close, high-quality relationships support cognitive health through sustained cognitive, emotional, and social engagement (Ma et al., 2024; Zhang et al., 2024). Overall, the qualitative findings highlight how the social and emotional dimensions of partnership, often missed in quantitative models, shape older adults’ interpretations of cognitive change.

A well-established literature suggests that engaging in CLA may buffer age-related cognitive decline (Mao et al., 2023; Park et al., 2014); however, CLA did not significantly moderate the relationship between age and SCD in the present study. This indicates that the CLA level did not alter the effect of age on cognitive concerns, suggesting that while CLA engagement is generally beneficial, its moderating role in the age–SCD relationship may not be consistent. One possible explanation may lie in the analytic assumption of linearity. CLA engagement was modeled as uniformly beneficial, yet the primary protective gain may occur when individuals move from low to moderate engagement, with limited additional benefit at higher levels. If CLA effects are not dose-dependent, moderation effects may be obscured in cross-sectional analyses. Another explanation relates to limited variability in engagement, as many participants were already regularly involved in CLA, potentially reducing statistical power to detect moderation. Additionally, CLA engagement may be beneficial over time in a manner that requires following individuals longitudinally, making any moderation effect difficult to detect cross-sectionally. This may reflect a cognitive reserve effect, whereby engagement in mentally stimulating activities provides resilience against age-related cognitive concerns that appear later on (Stern, 2009).

Recent neuroimaging evidence further contextualizes these findings. In a randomized controlled trial involving older adults with SCD, Lee et al. (2024) found that increased engagement in calligraphy strengthened functional connectivity within the default mode network, despite no measurable changes in global cognitive performance. This suggests that cognitive benefits of CLA may occur at the neural level that are not captured by standard SCD measures, highlighting the importance of multi-modal outcome assessment. Notably, neural connectivity deteriorated in the control group despite maintaining their usual level of activity, whereas increased practice led to improved connectivity in the intervention group (Lee et al., 2024). This implies that maintaining cognitive benefit may require increasing the frequency, intensity, or variety of activities, rather than simply sustaining habitual engagement. Consistently, participants in the present study reported perceiving both cognitive and emotional benefits from CLA, describing improvements in memory, attention, word retrieval, and confidence, alongside enhanced motivation to remain mentally active. While the moderating role of CLA on age-related SCD was not statistically significant, participants’ qualitative reports highlight perceived cognitive and emotional benefits, suggesting that even modest quantitative associations may reflect meaningful lived experiences. Thus, future studies should consider multiple outcomes, including neural and physiological markers, standard cognitive assessments, and subjective experiences, to more comprehensively capture the effects of CLA engagement.

Specific types of CLAs may differentially support cognitive domains, depending on their cognitive demand and social context. Socially interactive activities, such as spending time with friends or creative tasks, were linked to improvements in working memory and processing speed, whereas intellectually demanding pursuits, like reading, puzzles, or problem-solving, were associated with broader gains in executive function and orientation (Bielak et al., 2019; Fernández et al., 2023). In line with this, the current research found that engagement in puzzles, reading, and cultural events was associated with lower SCD concerns across memory, language, and executive domains. Nevertheless, qualitative data revealed that these same activities could also make participants more aware of decline, sometimes causing frustration when tasks felt overwhelming. These findings align with Cognitive Reserve (CR) theory, which posits that mentally stimulating activities enhance cognitive resilience, yet may also make subtle cognitive deficits more noticeable as individuals recruit cognitive resources to maintain performance (Stern, 2009). A similar dual effect was also reported by Pillai et al. (2011), who found that late-life crossword puzzle participation delayed the onset of accelerated memory decline in individuals who later developed dementia by approximately 2.5 years; once decline began, however, these individuals exhibited steeper deterioration than non-puzzlers. This mirrors the present qualitative accounts, where puzzles and reading were seen as both cognitively protective and emotionally challenging, illustrating how CR can delay observable decline while revealing subtle vulnerabilities.

### Implications for practice and research

Findings from this study underscore the need for interventions that address both cognitive and emotional aspects of SCD to support sustained engagement in CLA. Primary care providers, particularly general practitioners, should incorporate brief screenings for emotional distress, such as depression or anxiety, alongside cognitive assessments. This would help identify both underlying contributors to SCD and factors that may indirectly limit engagement in CLA, enabling timely referral and support. Flexible caregiver support, including respite services and tailored programs, remains essential, particularly given that older adults facing caregiving responsibilities or practical barriers tend to engage less in CLA, limiting opportunities for cognitive stimulation. Enhancing the visibility and accessibility of local CLA opportunities is also critical: older adults need up-to-date community listings, clear schedules, and public outreach campaigns about available activities, and mobility barriers should be addressed through transport services, volunteer ride-sharing, or in-home digital platforms. Researchers can also support the design, evaluation, and refinement of interventions to ensure they are practical, user-friendly, and widely adopted by older adults.

### Strengths and limitations

To our knowledge, this is the first mixed-methods study to comprehensively examine the relationship between SCD and CLA engagement across domain-specific cognitive concerns. Drawing on a recent systematic review (Akbayrak et al., 2024), the research addresses key gaps by extending beyond memory to include language and executive function and exploring how specific CLA types relate to these domains. It is also the first to qualitatively investigate older adults’ experiences, motivations, and emotional responses to CLA in the context of SCD, highlighting the complex and sometimes contradictory role of CLA in perceived cognitive changes. Methodological strengths include a sequential explanatory mixed-methods design, integrating robust quantitative associations with rich qualitative insights, and the use of validated multidimensional measures (SCD-Q for multiple cognitive domains; CLAS for diverse activity types). Mixed-mode recruitment (online and in-person) enhanced accessibility and diversity, including participants with limited mobility or digital confidence.

Despite these strengths, the study has limitations. Its cross-sectional design precludes causal interpretation of relationships between CLA and SCD, and longitudinal data are needed to clarify directionality and potential bidirectional influences. Convenience sampling (i.e., recruiting participants already attending activities) may limit generalizability, as these individuals were likely more cognitively active and health-literate than the broader older population. Men were underrepresented, which may constrain insights into gendered experiences of CLA and SCD. Additionally, while the CLAS was treated as continuous in analyses, it is ordinal, and subjective interpretation of frequency anchors (e.g., “several times per year”) may have introduced measurement error. Finally, reliance on self-reported cognitive concerns without objective assessment represents a key limitation: while participants were presumably free of diagnosed cognitive disorders, undiagnosed impairment cannot be entirely ruled out. Future research could incorporate brief objective cognitive measures to better distinguish SCD from early-stage cognitive disorders.

## Conclusion

This study provides novel, domain-specific insights into SCD and CLA engagement in older adults, highlighting patterns that may reflect reciprocal influences. Greater CLA participation was linked to lower SCD concerns, while cognitively demanding activities sometimes heightened awareness of decline yet also fostered cognitive abilities, emotional wellbeing, and social connectedness. Engagement was influenced by motivation, emotional responses, and contextual factors. Future research should adopt longitudinal designs, include diverse samples, and combine objective and subjective cognitive measures to clarify causal pathways and bidirectional effects. Promoting meaningful, accessible, socially supported CLA may enhance cognitive and emotional functioning, supporting person-centered strategies for maintaining cognitive health.

## Supplementary Material

igag088_Supplementary_Data

## Data Availability

The data that support the findings of this study are not publicly available due to privacy and ethical restrictions. Supplementary material contains all additional information necessary to support the findings. This study was not preregistered.
