# Supplementary material for: Subjective cognitive decline and cognitive leisure activity engagement in older adults: a mixed-methods study
Source: Innov Aging. 2026 Aug 6;10(11):igag088. doi: 10.1093/geroni/igag088 (PMC13695511; doi:10.1093/geroni/igag088)
Supplement: Supplementary file 1 [file igag088_supplementary_data.pdf]

***Innovation in Aging* Supplementary Material: Akbayrak, Powell, & Barnes. Subjective cognitive decline and cognitive leisure activity engagement in older adults: A mixed-methods study**

Contents

Supplementary Section 1: Semi-Structured Qualitative Interview Guide and Interview Schedule (Qualitative Phase) 2
Supplementary Section 2: Code Frequency Grid Demonstrating Theme and Subtheme Coverage Across Participants 4
Supplementary Section 3: Reporting Checklists for Quantitative, Qualitative, and Mixed Methods Components 5
Supplementary Section 4: Mann–Whitney U Test Results for Total and Domain-specific SCD Score by Gender, Education, and Marital Status 11
Supplementary Section 5: Domain-Specific Linear Regression Analyses Predicting Subjective Cognitive Decline 12
Supplementary Section 6: Moderation Analyses of Cognitive Leisure Activity on Age and Domain-Specific Subjective Cognitive Decline 14

Supplementary Section 1: Semi-Structured Qualitative Interview Guide and Interview Schedule (Qualitative Phase)

Interview Guide
This semi-structured interview guide was developed to explore participation in brain health activities (i.e., cognitive leisure activities, CLA) among older adults experiencing forgetfulness. The guide was used flexibly; not all questions were asked in every interview and probing or follow-up questions were used as appropriate to clarify or expand participants' responses.

Pre-Interview Procedures
Participants received an information sheet and provided informed consent prior to participation (written consent for face-to-face interviews and digital consent for online interviews). Participants were informed about the study aims, confidentiality, voluntary participation, and their right to withdraw at any time or skip questions they felt uncomfortable answering. With participants' permission, interviews were audio- or video-recorded.

Key Interview Questions
1. Can you describe any changes you have noticed in your memory or brain health over the past few years or months?
2. Do you feel concerned or anxious about your forgetfulness?
3. Did you consult a doctor or healthcare professional about these concerns?
4. What do you think are the barriers to engaging in brain health activities (cognitive leisure activities)?
5. What motivates you to participate in brain health activities?
6. Do you believe brain health activities have helped with forgetfulness? If so, in what ways?
7. What resources or support systems would you have liked to see for people experiencing memory problems?
8. What role do you think healthcare professionals, researchers, or community organisations should play in supporting older individuals with forgetfulness?
9. Is there anything else about your experiences with forgetfulness or brain health activities that you would like to share?

Interviews concluded by thanking participants for their time and reiterating confidentiality.

Table S1. Interview schedule

STEP	Phase	Key actions
**1**	Objectives	Exploring factors influencing participation in cognitive leisure activities (CLA) among individuals with subjective cognitive decline (SCD)
**2**	Introduction	Confirming that participants had read the Participant Information Sheet and provided informed consent; introducing the study purpose, confidentiality, right to withdraw, and interview procedures; commencing audio/video recording with permission
**3**	During the interview	Guiding participants through the interview questions and using follow-up prompts where necessary to clarify responses
**4**	Ending the interview	Ensuring all key topics were covered; thanking participants for their contribution; stopping the recording; providing information on relevant support services if required

Supplementary Section 2: Code Frequency Grid Demonstrating Theme and Subtheme Coverage Across Participants
This code frequency grid illustrates the distribution of identified themes and subthemes across participants, demonstrating the adequacy of data to support thematic analysis. Checkmarks indicate the presence of a given theme or subtheme within each participant's interview.

Table S2. Code frequency grid for thematic analysis across participants

Themes & Subthemes	P1	P2	P3	P4	P5	P6	P7	P8	P9	P10	P11	P12	P13	P14	P15	P16	P17	P18	P19	P20
**Theme 1: Motivations for Cognitive Leisure Activity**																				
**1. Enjoyment of cognitive leisure activities**	-	-	✅	-	-	✅	✅	✅	✅	✅	✅	✅	-	-	✅	✅	✅	✅	-	✅
**2. Preventing cognitive decline**																				
a) Maintaining cognitive and emotional well-being	✅	✅	-	-	✅	-	-	-	✅	-	-	✅	✅	✅	✅	-	-	-	✅	✅
b) Perceived benefits for cognitive health	-	✅	✅	-	-	-	✅	✅	-	✅	-	-	✅	✅	✅	-	✅	-	✅	✅
**3. Confidence and sense of achievement**	-	-	-	-	-	✅	✅	✅	-	✅	-	-	✅	-	✅	-	-	-	-	-
**4. Maintaining social engagement**	-	-	-	✅	-	-	✅	-	-	✅	-	-	-	-	-	✅	-	-	✅	-
**Theme 2: Barriers to Cognitive Leisure Activity**																				
**1. Perceived inability to engage in cognitive leisure activity**	✅	✅	-	-	✅	✅	-	✅	✅	-	✅	-	-	✅	-	✅	✅	✅	✅	-
**2. Psychological and emotional barriers**	-	-	-	-	✅	✅	✅	✅	✅	-	✅	-	✅	✅	✅	-	✅	✅	✅	-
**3. Health-related limitations**	-	✅	✅	-	✅	-	✅	-	-	-	-	-	-	-	✅	✅	-	✅	✅	-
**4. Time constraints and caregiving responsibilities**	-	✅	✅	✅	-	-	-	-	-	-	-	✅	-	-	-	-	-	-	-	-
**Theme 3: Strategies to support Cognitive Leisure Activity engagement**																				
1. **Availability, accessibility, and awareness of cognitive leisure activity**	-	✅	✅	-	✅	-	✅	-	-	✅	-	✅	-	✅	✅	-	✅	✅	✅	✅
**2. The role of social encouragement**	✅	-	✅	-	-	✅	✅	-	-	-	-	✅	✅	-	-	✅	-	✅	✅	✅
**3. Empathy and tailored professional guidance**	✅	✅	✅	-	-	-	-	-	✅	✅	✅	-	✅	✅	✅	-	-	✅	-	-

Note. P1–P20 represent individual interview participants.

Supplementary Section 3: Reporting Checklists for Quantitative, Qualitative, and Mixed Methods Components

Table S3.1 STROBE checklist for the quantitative component

**Strengthening the reporting of observational studies in epidemiology (STROBE)**	**Where addressed in the present study**
**Introduction**	
Background/rationale: Explain the scientific background and rationale for the investigation being reported	The scientific background and rationale for examining the association between subjective cognitive decline (SCD) and cognitive leisure activity (CLA) engagement were described in the Background and Objectives.
Objectives: State specific objectives, including any prespecified hypotheses	The research aimed to quantitatively examine the association between SCD and CLA engagement among older adults.
**Methods**	
Study design: Present key elements of study design early in the paper	A sequential explanatory mixed methods design was employed, with the quantitative component conducted first (QUAN → QUAL).
Setting - Describe the setting, locations, and relevant dates, including periods of recruitment, exposure, follow-up, and data collection	A convenience sampling strategy was used between January and July 2024, with data collected via online (Qualtrics) and in-person recruitment in the United Kingdom (UK).
Participants - Give the eligibility criteria, and the sources and methods of selection of participants	Participants were community-dwelling adults aged ≥60 years, residing in the UK, without diagnosed mental health conditions (e.g., depression, schizophrenia, anxiety) or neurocognitive disorders (e.g., dementia or Alzheimer's disease).
Variables - Clearly define all outcomes, exposures, predictors, potential confounders, and effect modifiers. Give diagnostic criteria, if applicable	SCD concerns were defined as the outcome variable, while CLA engagement was the primary exposure variable. Sociodemographic characteristics (age, gender, education, marital status) were considered as potential predictors or covariates.
* Data sources/ measurement - For each variable of interest, give sources of data and details of methods of assessment (measurement). Describe comparability of assessment methods if there is more than one group	Data were collected using a structured survey including sociodemographic information, the Subjective Cognitive Decline Questionnaire (SCD-Q), and the Cognitive Leisure Activity Scale (CLAS).
Bias - Describe any efforts to address potential sources of bias	Potential selection bias was mitigated through combined online and paper-based recruitment. Response bias was addressed by ensuring anonymity, confidentiality, and standardised survey instructions.
Study size - Explain how the study size was arrived at	Sample size was determined a priori using G*Power, based on a small effect size (f^2 = 0.048), α = 0.05, power = 0.80, and five predictors, yielding a minimum required sample of 166 participants.
Quantitative variables - Explain how quantitative variables were handled in the analyses. If applicable, describe which groupings were chosen and why	SCD total and domain-specific scores were treated as continuous variables. Overall CLA engagement was analysed as a continuous variable, while individual CLA activities (originally measured on a 6-point Likert scale) were recoded as binary indicators (0 = no engagement; 1 = any engagement). Age was treated as continuous, and education, marital status, and gender were coded as binary categorical variables.

**Strengthening the reporting of observational studies in epidemiology (STROBE)**	**Where addressed in the present study**
Statistical methods<br>(a) Describe all statistical methods, including those used to control for confounding<br>(b) Describe any methods used to examine subgroups and interactions<br>(c) Explain how missing data were addressed<br>(d) If applicable, describe analytical methods taking account of sampling strategy<br>(e) Describe any sensitivity analyses	Analyses included data screening and reliability checks, descriptive statistics, normality testing, Spearman correlations, Mann–Whitney U tests, linear regression, and moderated regression analyses to examine associations and interactions while controlling for confounders.
**Results/Findings**	
* Participants<br>a) Report numbers of individuals at each stage of study—e.g. numbers potentially eligible, examined for eligibility, confirmed eligible, included in the study, completing follow-up, and analysed<br>b) Give reasons for non-participation at each stage<br>c) Consider use of a flow diagram	The final sample consisted of 255 older adults (mean age = 76.9 years, *SD* = 8.2), of whom 74.9% were women. Based on SCD-Q scores, 51% of participants were classified as having SCD.
* Descriptive data<br>a) Give characteristics of study participants (e.g. demographic, clinical, social) and information on exposures and potential confounders<br>b) Indicate number of participants with missing data for each variable of interest	Participant characteristics (age, gender, education, marital status), SCD scores (total and domain-specific), and CLA engagement (total score and activity variety) were summarised using means, standard deviations, ranges, and percentages. No missing data were observed.
* Outcome data - Report numbers of outcome events or summary measures	SCD outcomes were reported as continuous total and domain-specific scores (memory, language, executive function). CLA engagement was reported both continuously (total score and variety) and categorically (binary indicators for individual activities).
Main results<br>a) Give unadjusted estimates and, if applicable, confounder-adjusted estimates and their precision (e.g., 95% confidence interval). Make clear which confounders were adjusted for and why they were included<br>b) Report category boundaries when continuous variables were categorized<br>c) If relevant, consider translating estimates of relative risk into absolute risk for a meaningful time period	Unadjusted analyses indicated small but significant negative associations between CLA engagement and SCD scores, and positive associations between age and SCD. Linear regression models adjusted for age showed that higher CLA engagement predicted lower SCD scores, while age predicted higher SCD scores. 95% bootstrapped confidence intervals supported estimate precision. Sociodemographic variables (gender, education, marital status) were tested but not retained in adjusted models due to non-significance.
Other analyses - Report other analyses done—e.g. analyses of subgroups and interactions, and sensitivity analyses	NA
**Discussion**	
Key results - Summarise key results with reference to study objectives	Discussion and Implications: Findings were discussed in relation to existing literature, highlighting the role of CLA in SCD and practical implications for older adults.<br>Strengths and Limitations: Key methodological strengths and limitations, including potential biases,
Limitations - Discuss limitations of the study, taking into account sources of potential bias or imprecision. Discuss both direction and magnitude of any potential bias	

Strengthening the reporting of observational studies in epidemiology (STROBE)	Where addressed in the present study
Interpretation - Give a cautious overall interpretation of results considering objectives, limitations, multiplicity of analyses, results from similar studies, and other relevant evidence	were acknowledged to provide context for interpreting the results.<br><br>Conclusion: The study's original contributions and suggestions for future research directions were summarized.
Generalisability - Discuss the generalisability (external validity) of the study results	
Other information	
Funding - Give the source of funding and the role of the funders for the present study and, if applicable, for the original study on which the present article is based	NA

*Give information separately for exposed and unexposed groups.

Table S3.2 SRQR checklist for the qualitative component

Standards for Reporting Qualitative Research (SRQR)	Where addressed in this research or explanation
**Introduction**	
Problem formulation - Description and significance of the problem/phenomenon studied; review of relevant theory and empirical work; problem statement	The background and significance of the research were described in the Background and Objectives.
Purpose or research question - Purpose of the study and specific objectives or questions	The qualitative phase aimed to explore older adults' experiences and the factors influencing engagement in cognitive leisure activities (CLA) in the context of subjective cognitive decline (SCD), including, but not limited to, motivations and barriers.
**Methods**	
Qualitative approach and research paradigm - Qualitative approach (e.g., ethnography, grounded theory, case study, phenomenology, narrative research) and guiding theory if appropriate; identifying the research paradigm (e.g., postpositivist, constructivist/ interpretivist) is also recommended; rationale**	The qualitative phase employed a coding reliability approach to thematic analysis within a pragmatic paradigm. Thematic domains were determined a priori based on the research aims, consistent with the postpositivist quality standards underpinning this analytic approach.
Researcher characteristics and reflexivity - Researchers' characteristics that may influence the research, including personal attributes, qualifications/experience, relationship with participants, assumptions, and/or presuppositions; potential or actual interaction between researchers' characteristics and the research questions, approach, methods, results, and/or transferability	The first author's background in gerontology and experience as a non-native English speaker were recognised as potential sources of bias in data collection and interpretation. These were managed through regular supervision, independent coding by multiple researchers, and consensus-based discussion, ensuring balanced and transparent analysis.
Context - Setting/site and salient contextual factors; rationale**	Participants were older adults (≥60 years) involved in charitable and community organizations across the UK. These organizations were selected due to their extensive outreach and engagement with the older adult demographic, which aligns with the study's focus on this group.
Sampling strategy - How and why research participants, documents, or events were selected; criteria for deciding when no further sampling was necessary (e.g., sampling saturation); rationale**	Purposive sampling was used to select participants with SCD (SCD-Q ≥7), ensuring variation in both SCD severity and CLA engagement levels. A code frequency grid was used to track when no new codes

Standards for Reporting Qualitative Research (SRQR)	Where addressed in this research or explanation
	emerged across interviews, consistent with coding reliability thematic analysis (see Supplementary File 2).
Ethical issues pertaining to human subjects - Documentation of approval by an appropriate ethics review board and participant consent, or explanation for lack thereof; other confidentiality and data security issues	Ethical approval was obtained from the University of Sheffield. Participants provided informed consent. Confidentiality and data security were maintained.
Data collection methods - Types of data collected; details of data collection procedures including (as appropriate) start and stop dates of data collection and analysis, iterative process, triangulation of sources/methods, and modification of procedures in response to evolving study findings; rationale**	Semi-structured interviews were conducted between September–November 2024, face-to-face or online. Interviews were audio-recorded. Procedures were adapted iteratively based on preliminary findings.
Data collection instruments and technologies - Description of instruments (e.g., interview guides, questionnaires) and devices (e.g., audio recorders) used for data collection, if/how the instrument(s) changed over the course of the study	Interviews followed a flexible guide developed from literature and quantitative findings. Audio recordings were used for transcription; the guide was refined during data collection to incorporate emerging insights.
Units of study - Number and relevant characteristics of participants, documents, or events included in the study; level of participation (could be reported in results)	20 participants (4 male, 16 female), aged 60–85, participated.
Data processing - Methods for processing data prior to and during analysis, including transcription, data entry, data management and security, verification of data integrity, data coding, and anonymization/de-identification of excerpts	Interviews were transcribed and imported into NVivo. Coding was iterative, verified, and anonymized to ensure data integrity.
Data analysis - Process by which inferences, themes, etc., were identified and developed, including the researchers involved in data analysis; usually references a specific paradigm or approach; rationale**	Interview data were analysed using a coding reliability approach to thematic analysis. Full details of the analytic approach, including the rationale for method selection, coding procedures, and intercoder agreement process, are provided in the Methods-Analysis section of the main manuscript.
Techniques to enhance trustworthiness - Techniques to enhance trustworthiness and credibility of data analysis (e.g., member checking, audit trail, triangulation); rationale**	Trustworthiness was established through procedures consistent with the quality standards of coding reliability thematic analysis. Coding quality was demonstrated through the use of multiple independent coders, subjective intercoder agreement, and consensus-based resolution of coding discrepancies, which served to manage researcher subjectivity as a potential source of bias. The finalised coding structure was applied consistently across all transcripts. Transferability was supported through purposive sampling to ensure variation in SCD severity and CLA engagement levels, alongside rich contextual descriptions of the sample and setting.
**Results/Findings**	
Synthesis and interpretation - Main findings (e.g., interpretations, inferences, and themes); might include development of a theory or model, or integration with prior research or theory	Key themes and subthemes on motivations, barriers, and strategies for CLA engagement were identified, illustrated with participant quotes, and integrated with quantitative findings via joint displays.

Standards for Reporting Qualitative Research (SRQR)	Where addressed in this research or explanation
Links to empirical data - Evidence (e.g., quotes, field notes, text excerpts, photographs) to substantiate analytic findings	Participant quotes were provided to substantiate themes and interpretations.
**Discussion**	
Integration with prior work, implications, transferability, and contribution(s) to the field - short summary of main findings; explanation of how findings and conclusions connect to, support, elaborate on, or challenge conclusions of earlier scholarship; discussion of scope of application/generalizability; identification of unique contribution(s) to scholarship in a discipline or field	Findings were integrated with quantitative results, highlighting patterns suggesting potential reciprocal influences between CLA engagement and SCD. Limitations, including the cross-sectional design, potential self-selection bias, and the absence of objective cognitive assessments, were discussed.
Limitations - Trustworthiness and limitations of findings	Trustworthiness was addressed through methodological rigor, but sample characteristics (e.g., underrepresentation of males) may affect generalisability.
**Other**	
Conflicts of interest - Potential sources of influence or perceived influence on study conduct and conclusions; how these were managed	None
Funding - Sources of funding and other support; role of funders in data collection, interpretation, and reporting	NA

**The rationale should briefly discuss the justification for choosing that theory, approach, method, or technique rather than other options available, the assumptions and limitations implicit in those choices, and how those choices influence study conclusions and transferability. As appropriate, the rationale for several items might be discussed together.

Table S3.3 GRAMMS checklist for mixed methods research

GRAMMS Criterion	Application in this research
Describe the justification for using a mixed methods approach to the research question	A mixed methods design enabled comprehensive understanding of the relationship between subjective cognitive decline (SCD) and cognitive leisure activity (CLA) engagement. Justification included: complementarity (qualitative insights explain quantitative patterns), development (quantitative results informed interview design), initiation (generate new interpretations), expansion (address different aspects with each method), and triangulation (corroborate findings).
Describe the design in terms of the purpose, priority, and sequence of methods	A sequential explanatory design (QUAN → QUAL) was employed. Quantitative analyses identified statistical associations, followed by qualitative interviews to explore underlying factors. Both strands were given equal methodological weight to provide complementary insights.
Describe each method in terms of sampling, data collection and analysis	Quantitative phase: Convenience sample of 255 adults (≥60 years) in the United Kingdom (UK) via online and in-person recruitment. Data: The Subjective Cognitive Decline Questionnaire (SCD-Q) and the Cognitive Leisure Activity Scale (CLAS); analyses: descriptive statistics, correlations, linear and moderated regressions.<br><br>Qualitative phase: 20 purposively sampled participants with SCD (SCD-Q ≥7), representing high/low CLA engagement. Semi-structured interviews conducted in person or online; analysed thematically.
Describe where integration has occurred, how it has occurred and who has participated in it	Integration occurred at multiple stages. Quantitative findings informed purposive sampling and interview guide development (design-level integration). Joint displays were used to synthesize quantitative and qualitative results (interpretive-level integration).
Describe any limitation of one method associated with the presence of the other method	While the mixed methods design provided comprehensive insights into the relationship between SCD and CLA engagement, it also posed challenges. The sequential explanatory approach extended the overall study timeline and required careful coordination between phases. Additionally, balancing equal methodological weight between quantitative and qualitative strands demanded rigorous planning to ensure neither was underemphasized.
Describe any insights gained from mixing or integrating methods	Integration revealed not only statistical associations but also underlying motivations and barriers. Qualitative data explained quantitative patterns, informing actionable strategies for enhancing CLA participation. These insights have implications for community programs, healthcare professionals, and policymakers.

Supplementary Section 4: Mann–Whitney U Test Results for Total and Domain-specific SCD Score by Gender, Education, and Marital Status

This section presents the Mann–Whitney *U* test results examining differences in total and domain-specific subjective cognitive decline (SCD) score (memory, language, and executive function) by gender, education level, and marital status. As shown in **Table S4.1**, no statistically significant differences were observed in total SCD score or in any of the domain-specific scores across gender, education, or marital status.

Non-parametric Mann–Whitney *U* tests were applied across all comparisons and effect sizes (Cohen's *d*) were calculated for all outcomes following a two-step procedure. First, the rank-biserial correlation (*r*) was derived by dividing the standardized test statistic (z) from the Mann–Whitney *U* test by the square root of the total sample size (Tomczak & Tomczak, 2014). Second, this correlation coefficient was converted into Cohen's *d* to facilitate interpretation and comparison with parametric effect sizes (Borenstein, 2009). Across all analyses, effect sizes were minimal and consistent with the non-significant results.

Table S4.1. SCD Score by Education Level, Marital Status, and Gender

Domain	Variable	Mann-Whitney *U*	*p*	Cohen's *d*
**Total SCD**	Gender	6036.50	.88	.019
	Education level	5030.50	.84	.025
	Marital Status	7841.00	.92	.014
**SCD-Memory**	Gender	6092.50	.97	.005
	Education level	4976.00	.75	.004
	Marital Status	7800.00	.87	.024
**SCD-Executive Function**	Gender	5628.50	.33	.121
	Education level	4902.00	.63	.006
	Marital Status	7862.50	.95	.008
**SCD-Language**	Gender	5996.50	.819	.003
	Education level	5029.00	.835	.003
	Marital Status	*U* =7809.50	.879	.002

Note. SCD = subjective cognitive decline

Supplementary Section 5: Domain-Specific Linear Regression Analyses Predicting Subjective Cognitive Decline

The supplementary tables present detailed domain-specific linear regression analyses examining the relationships between cognitive leisure activity (CLA), age, and subjective cognitive decline (SCD). CLA engagement variety was excluded from the regression models due to its high correlation with CLA engagement score (r = .823, p < .01), which raised concerns about multicollinearity. Age was included as a covariate because it showed a significant bivariate relationship with total SCD scores, whereas other demographic variables did not. To ensure robustness of parameter estimates and to address potential non-normality in residuals, percentile bootstrapping with 1,000 resamples was applied to generate 95% confidence intervals. B, Standard Error, Beta, *t*, and *p*-values are reported from the standard regression coefficients table, whereas 95% confidence intervals were derived using percentile bootstrapping with 1,000 resamples. Effects were interpreted as statistically robust if the bootstrapped confidence intervals did not include zero (Efron, 1993; Field, 2024).

The regression model predicting memory score was statistically significant, F (2, 252) = 7.362, p < .001, with an adjusted R^2 of .048 (see **Table S5.1)**. Age significantly predicted memory score (b = .044, β = .138, p = .026, 95% CI [.00, .09]), indicating that older individuals reported greater memory-related concerns. Similarly, CLA engagement frequency was a significant negative predictor of memory score (b = -.052, β = -.181, p = .003, 95% CI [-.09, -.02]), suggesting that higher engagement in CLA was associated with fewer memory complaints.

Table S5.1. Linear Regression Predicting Memory Score

Variable	*b*	*SE*	*β*	*t*	*p*	95% CI
**Constant**	2.698	.159	–	16.966	< .001	[2.39, 3.01]
**Age**	.044	.019	.138	2.246	.026	[.00, .09]
**CLA Engagement Frequency**	-.052	.018	-.181	-2.949	.003	[-.09, -.02]
**Model Summary**	R^2 = .055, Adjusted R^2 = .048<br>F (2, 252) = 7.362, p < .001					

Note. 95% confidence intervals (CI) calculated using 1000 bootstrap samples; CLA = cognitive leisure activity; SCD = subjective cognitive decline

The regression model predicting language score was statistically significant, F (2, 252) = 5.573, p = .004, with an adjusted R^2 of .035 (see **Table S5.2**). CLA engagement frequency score significantly and negatively predicted language score (b = -.032, β = -.162, p = .009, 95% CI [-.06, -.01]), suggesting that greater CLA engagement frequency was associated with fewer language-related cognitive concerns. Age was a marginally significant positive predictor (b = .025, β = .116, p = .061, 95% CI [.00, .06]), indicating a trend toward increased language-related complaints with advancing age.

Table S5.2. Linear Regression Predicting Language Score

Variable	*b*	*SE*	*β*	*t*	*p*	95% CI
**Constant**	2.442	.110	–	22.185	< .001	[2.21, 2.66]
**Age**	.025	.013	.116	1.881	.061	[.00, .06]
**CLA Engagement Frequency**	-.032	.012	-.162	-2.622	.009	[-.06, -.01]
**Model Summary**	R^2 = .042, Adjusted R^2 = .035<br>F (2, 252) = 5.573, p = .004					

Note. 95% confidence intervals (CI) calculated using 1000 bootstrap samples; CLA = cognitive leisure activity; SCD = subjective cognitive decline

The regression model predicting executive function score was statistically significant, F (2, 252) = 8.656, $p < .001$, with an adjusted R^2 of .057 (see **Table S5.3**). Age significantly predicted executive function score (b = .045, $β$ = .191, p = .002, 95% CI [.02, .08]), indicating that older individuals reported more executive-related cognitive concerns. CLA engagement frequency also significantly and negatively predicted executive function score (b = -.033, $β$ = -.155, p = .012, 95% CI [-.06, -.09]), suggesting that greater engagement in CLA was associated with fewer executive complaints.

Table S5.3. Linear Regression Predicting Executive Function Score

Variable	*b*	*SE*	*β*	*t*	*p*	95% CI
**Constant**	2.042	.119	–	17.201	< .001	[1.80, 2.29]
**Age**	.045	.015	.191	3.120	.002	[.02, .08]
**CLA Engagement Frequency**	-.033	.013	-.155	-2.531	.012	[-.06, -.09]
**Model Summary**	R^2 = .064, Adjusted R^2 = .057<br>F (2, 252) = 8.656, p < .001					

Note. 95% confidence intervals (CI) calculated using 1000 bootstrap samples; CLA = cognitive leisure activity; SCD = subjective cognitive decline

Supplementary Section 6: Moderation Analyses of Cognitive Leisure Activity on Age and Domain-Specific Subjective Cognitive Decline

The supplementary tables present detailed domain-specific moderation analyses testing whether cognitive leisure activity (CLA) engagement moderates the association between age and domain-specific subjective cognitive decline (SCD) concerns.

Hierarchical moderated regression analyses were conducted for all three SCD domains (memory, language, and executive function) to examine whether CLA engagement frequency moderated the relationship between age and domain-specific SCD scores. Age was selected as the predictor variable based on prior analyses, which showed significant bivariate and multivariate associations with SCD scores, whereas other demographic variables (e.g., gender, education level, marital status) did not. All continuous predictor variables were mean-centred prior to computing the interaction term between age and CLA engagement frequency. To ensure robustness of parameter estimates and to address potential non-normality in residuals, percentile bootstrapping with 1,000 resamples was applied to generate 95% confidence intervals. B, Standard Error, Beta, t, and p-values are reported from the standard regression coefficients table, whereas 95% confidence intervals were derived using percentile bootstrapping.

Subjective cognitive decline-Memory

In Step 1, age and CLA engagement were entered as predictors, explaining a significant proportion of variance in memory SCD score ($R^2 = .055$, F (2,252) = 7.362, $p < .001$). Both CLA engagement ($\beta = -.181$, $p = .003$) and age ($\beta = .138$, $p = .026$) emerged as significant predictors, indicating that higher CLA engagement was associated with fewer memory concerns, while older age predicted greater concerns. In Step 2, the interaction term (Age × CLA engagement) was added, but the change in explained variance was not significant ($\Delta R^2 = .001$, ΔF (1,251) = .141, $p = .708$), suggesting that the relationship between age and memory-related SCD concerns was not significantly moderated by CLA engagement frequency.

Table S6.1. Moderation Analysis Predicting Memory Score

Variable	*b*	*SE*	*β*	*t*	*p*	95% CI
**Model 1**						
**Constant**	2.698	.159	-	16.966	< .001	[2.40, 2.99]
**Age**	.044	.019	.138	2.246	.026	[-.00, .09]
**CLA Engagement Frequency**	-.052	.018	-.181	-2.949	.003	[-.09, -.02]
**Model Summary**	$R^2 = .055$, Adjusted $R^2 = .048$					
	F (2, 252) = 7.362, $p < .001$					
**Model 2**						
**Constant**	2.703	.160	-	16.917	< .001	[2.41, 3.00]
**Age**	.045	.020	.141	2.272	.024	[.00, .09]
**CLA Engagement Frequency**	-.053	.018	-.183	-2.967	.003	[-.09, -.02]
**CLA Engagement Frequency x Age**	.001	.002	.023	.376	.708	[-.00, .01]
**Model Summary**	$R^2 = .056$, $\Delta R^2 = .001$					
	F (3, 251) = 4.939, $p = .002$					
	ΔF (1,251) = .141, $p = .708$					

Note. 95% confidence intervals (CI) calculated using 1000 bootstrap samples; CLA = cognitive leisure activity

Subjective cognitive decline-Language

In Step 1, age and CLA engagement were entered as predictors, explaining a modest but significant portion of variance in language SCD score (R^2 = .042, F (2,252) = 5.573, p = .004). CLA engagement emerged as a significant predictor (β = -.162, p = .009), while age was marginally significant (β = .116, p = .061). In Step 2, the interaction term (Age × CLA engagement) was added, but it did not significantly increase explained variance (ΔR^2 = .002, ΔF (1,251) = .471, p = .493), indicating that CLA engagement did not moderate the relationship between age and language-related SCD concerns.

Table S6.2. Moderation Analysis Predicting Language Score

Variable	*b*	*SE*	*β*	*t*	*p*	95% CI
**Model 1**						
**Constant**	2.442	.110	-	22.185	< .001	[2.23, 2.66]
**Age**	.025	.013	.116	1.881	.061	[.00, .05]
**CLA Engagement Frequency**	-.032	.012	-.162	-2.622	.009	[-.06, -.01]
**Model Summary**	R^2 = .042, Adjusted R^2 = .035<br>F (2, 252) = 5.573, p = .004					
**Model 2**						
**Constant**	2.448	.111	-	22.150	< .001	[2.23, 2.67]
**Age**	.027	.014	.122	1.953	.052	[.00, .05]
**CLA Engagement Frequency**	-.033	.012	-.166	-2.674	.008	[-.06, -.01]
**CLA Engagement Frequency x Age**	.001	.002	.043	.687	.493	[-.00, .00]
**Model Summary**	R^2 = .044, ΔR^2 = .002<br>F (3, 251) = 3.865, p = .010<br>ΔF (1,251) = .471, p = .493					

Note. 95% confidence intervals (CI) calculated using 1000 bootstrap samples; CLA = cognitive leisure activity

Subjective cognitive decline-Executive Function

In Step 1, age and CLA engagement were entered as predictors, explaining 6.4% of variance in executive SCD score (R^2 = .064, F (2,252) = 8.656, p < .001). Both age (β = .191, p = .002) and CLA engagement (β = -.155, p = .012) were significant predictors, indicating that higher CLA engagement predicted lower executive SCD concerns, while older age predicted greater concerns. In Step 2, the interaction term (Age × CLA engagement) was added, but it did not significantly increase explained variance (ΔR^2 = .002, ΔF (1,251) = .421, p = .517), indicating that CLA engagement did not significantly moderate the relationship between age and executive function-related SCD concerns.

Table S6.3. Moderation Analysis Predicting Executive Function Score

Variable	*b*	*SE*	*β*	*t*	*p*	95% CI
**Model 1**						
**Constant**	2.042	.119	-	17.201	< .001	[1.82, 2.28]
**Age**	.045	.015	.191	3.120	.002	[.02, .07]
**CLA Engagement Frequency**	-.033	.013	-.155	-2.531	.012	[-.06, -.01]
**Model Summary**	R^2 = .064, Adjusted R^2 = .057<br>F (2, 252) = 8.656, p < .001					
**Model 2**						
**Constant**	2.048	.119	-	17.181	< .001	[1.82, 2.28]
**Age**	.047	.015	.196	3.175	.002	[.02, .08]
**CLA Engagement Frequency**	-.034	.013	-.159	-2.580	.010	[-.06, -.01]
**CLA Engagement Frequency x Age**	.001	.002	.040	.649	.517	[-.00, .01]
**Model Summary**	R^2 = .066, ΔR^2 = .002<br>F (3, 251) = 5.898, p < .001<br>ΔF (1,251) = .421, p = .517					

Note. 95% confidence intervals (CI) calculated using 1000 bootstrap samples; CLA = cognitive leisure activity
